# Editorial: New insights into inflammation driven autoimmune skin disorders: trends and challenges

**DOI:** 10.3389/fimmu.2026.1920024

**Published:** 2026-07-07

**Authors:** Manoj Kumar Tembhre, Alpana Sharma, Pankaj Dipankar, Shanid Mohiyuddin, Mohammad Tarique, Suneel Kumar

**Affiliations:** 1 Cardiac Biochemistry, All India Institute of Medical Sciences, New Delhi, India; 2 Development Research Division, Indian Council of Medical Research, New Delhi, India; 3 Department of Biochemistry, All India Institute of Medical Sciences (AIIMS), New Delhi, India; 4 National Institute of Nursing Research, National Institutes of Health, Bethesda, MD, United States; 5 Department of Medicine, University of Missouri, Columbia, MO, United States; 6 Department of Child Health, University of Missouri, Columbia, MO, United States; 7 Department of Biomedical Engineering, Rutgers, The State University of New Jersey, Piscataway, NJ, United States

**Keywords:** alopecia areata, autoimmunity, immunotherapies, inflammation, pemphigus vulgaris, primary lymphocytic scarring alopecias, psoriasis, systemic sclerosis

Skin is the dynamic organ where immune surveillance is constantly maintained by both innate and adaptive components of the immune system. However, it also harbors immune-privileged sites like the hair follicle bulb/bulge region, which is an active site of growth and has a niche for stem cells. Deregulation of fine-tuning between immune cells and the integumentary system often leads to the development of various inflammatory and autoimmune skin conditions like vitiligo, psoriasis, alopecia areata, systemic sclerosis, pemphigus vulgaris, etc. Despite extensive research in understanding the inherent biological processes involved in the pathogenesis of various skin diseases, critical gaps remain due to the involvement of complex networks, limiting the development of potential strategies to counteract the development and progression of these skin conditions. Immunotherapies have emerged as one of the promising treatment strategies, but have certain limitations. This editorial will provide insight into the innate and adaptive immune system and their crosstalk with skin-resident cells in various skin conditions, with emphasis on promising immunotherapies.

Keratinocytes act as an active orchestrator of epithelial immunity rather than acting as a passive structural component of skin and mucosa. Klimitz et al. systematically reviewed six major dermatological conditions, including psoriasis, lichen planus, atopic dermatitis, bullous pemphigoid, lupus erythematosus, and graft versus host disease (GVHD), and identified the function of keratinocytes beyond physical barrier. Keratinocytes are immunologically active sentinels involved in cytokine production, antigen presentation, and recruitment of other immune cells, shaping the immune microenvironment. Depending on the disease condition, they express various stress keratins and are also involved in signaling pathways like JAK-STAT, NF-κB, etc. The review highlights the significant role of keratinocytes in shaping the immune axis, such as Th2 dominance in atopic dermatitis and bullous pemphigoid and Th17 responses in psoriasis. Keratinocytes are also actively involved in crosstalk with immune cells like mast cells, T cells, macrophages, and NK cells. Furthermore, Hooda et al. demonstrated that pemphigus vulgaris (PV) is driven not only by desmosomal autoantibodies but also by profound innate immune dysregulation. Study revealed the critical involvement of NK cells - macrophages axis in driving the pathogenesis of PV. Hyperactivated NK cells state in PV through heightened cytotoxicity and IFN-γ secretion along with surface markers such as NKG2D drives the dysregulation of M2 macrophages, facilitating the upregulation of macrophages in PV. However, macrophage interplay with NK cells through the NKG2D-MICA/B axis promoted M1 polarization while impairing the M2 state. Additionally, NKG2D blockade revealed partial reversal of the inflammatory state, suggesting an active role of NKG2D and involvement of other contributors in this axis. Modulation of signaling pathways, such as those involved in keratinocytes and cellular cross-talks as seen in pemphigus vulgaris, provides promising approaches for future therapeutics. Targeting multicellular networks involved in pathogenesis may offer superior and effective therapeutics compared to singular targeting.

Vitiligo is another skin condition that is considered an autoimmune depigmentary disease where melanocytes are selectively destroyed by cytotoxic CD8^+^ T cells due to a deficiency of regulatory T cells (1, 2; Sharma et al.). Luo et al. described the advancement in the vitiligo treatment from the conventional immunosuppressants (e.g., corticosteroids and phototherapy) to next-generation targeted immunotherapies, where JAK inhibitors (e.g., ruxolitinib) were FDA approved for vitiligo in 2022 for topical use, whereas other oral JAK inhibitors (e.g., tofacitinib, baricitinib, upadacitinib) are undergoing trials for non-segmental active vitiligo. Further, systemic sclerosis (SSc) is a chronic autoimmune disease characterized by marked vasculopathy and tissue fibrosis. Liu described that the advancement of multi-omics techniques like single-cell, spatial transcriptomics, RNA sequencing, and proteomics has revealed various cell-specific therapeutic targets in various human diseases, including SSc. As a consequence of this, there is a paradigm shift in the treatment strategies that is more inclined towards targeted immunotherapies instead of generalized immunosuppression offered by conventional therapies. The emerging therapies include B-cell depletion (rituximab, belimumab), T-cell inhibition (abatacept), multi-cytokine targeting JAK inhibitors (e.g., tofacitinib, baricitinib), IL-6 inhibition (tocilizumab), and cell-based therapies (e.g., autologous and allogeneic CD19 CAR T cells). Immunotherapies may be instrumental in the treatment of autoimmune diseases like vitiligo and SSc treatment provided identification of candidate immune biomarkers that may directly drive the disease pathogenesis.

The role of neutrophil, particularly neutrophil extracellular traps (NETs), has garnered significant attention in the past two decades, and its role has been implicated in various autoimmune and inflammatory diseases, including psoriasis (3). Presence of NETotic neutrophils has been reported in lesional epidermis (Munro’s microabscesses) and dermis of psoriasis. The NETs armamentarium consists of potent inflammatory components like myeloperoxidases (MPO), neutrophil elastase (NE), proteinase 3 (PR3), cathepsin, LL-37, and β-defensin that can exacerbate the disease activity, leading to episodic appearance of disease flares. Tang et al. reported promising therapeutics against the NETs-derived components like NE, MPO, PAD4 (Peptidyl arginine deiminase 4, a key enzyme in NETosis), deoxyribonucleic acid (e.g., DNase I), and reactive oxygen species (ROS), where ROS scavengers (N-acetylcysteine (NAC), MitoTempo, ethyl pyruvate, diphenyleneiodonium, etc.) may play a significant role in neutrophil-driven dermatoses.

Over the past decade, the selective targeting of the IL-23 p19 subunit has emerged as one of the most significant developments in reshaping psoriasis treatments. A meta-analysis by Ashraf et al. presents the first comprehensive evaluation of mirikizumab (LY3074828) by pooling data from three randomized controlled trials involving 1,752 participants and demonstrating significant improvements in skin clearance rates compared with placebo. Importantly, therapeutic benefits extended beyond skin clearance to include nail disease, palmoplantar involvement, quality-of-life measures, and overall disease burden. Also, there were no significant differences in serious adverse events between groups, which supports the mechanistic rationale for selective p19 blockade over broader cytokine inhibition. Taken together, these findings suggest that mirikizumab is an effective IL-23 inhibitor, but with some limitations, including the lack of long-term data and head-to-head comparisons with established biologics, which were excluded from this meta-analysis. Complementing this therapeutic perspective, Jirouš Drulak et al. provide the first integrated characterization of γδ T cells in psoriasis vulgaris using various molecular techniques, including TCR sequencing, bulk transcriptomics, and flow cytometry, across 65 psoriatic vulgaris patients and 35 healthy controls. Disease severity and advancing age independently drove the contraction of peripheral γδ TCR diversity, with the loss of rare clonotypes and compensatory expansion of the TRGV9 and TRDV2 subsets, which are dynamically absent in healthy individuals. Transcriptomic profiling revealed a hyperactivated, cytotoxic, tissue-homing effector phenotype that closely recapitulates NK and CD8^+^ αβ effector cell signatures within psoriatic lesions. The identification of a transcriptionally distinct patient subgroup exhibiting amplified immune activation unexplained by clinical parameters raises the important possibility of immunological endotypes predictive of differential biologic responsiveness. Moreover, Rivera-Ruiz et al. study reported the first systematic transcriptomic meta-analysis in primary lymphocytic scarring alopecias, integrating six batch-corrected datasets from 134 patients across frontal fibrosing alopecia (FFA), lichen planopilaris (LPP), and central centrifugal cicatricial alopecia (CCCA), and 49 healthy controls. The three subtypes converge on a shared IFN-γ-driven inflammatory core but diverge considerably beyond it. FFA and LPP show pronounced JAK-STAT enrichment, with partial reversal achievable using brepocitinib. CCCA follows a different course, such as mitochondrial dysfunction, dominating epigenetic repression, and inhibition of the JAK pathway, as have a lower effect. This clear clinical distinction reveals that CCCA may require metabolically targeted intervention rather than the anti-inflammatory approaches effective in FFA and LPP. Importantly, downregulated gene sets enriched for cardiometabolic traits were observed across all three subtypes, suggesting that chronic scalp inflammation may have systemic consequences warranting closer comorbidity screening. In conclusion, endotype-based treatment selection is increasingly supported by direct evidence rather than theoretical rationale. Fibrosis continues to represent the principal barrier to recovery in both systemic sclerosis and scarring alopecia, and the repeated failure of anti-inflammatory therapy to reverse the established matrix remodeling indicates that intervention timing, not agent selection, may be the limiting factor. Earlier, pre-fibrotic treatment windows may therefore be more consequential than further refinement of existing anti-inflammatory regimens. Finally, single-cell and spatial multi-omics, integrated with artificial intelligence-based predictive modeling, present a credible though still unproven path toward disease modification rather than symptomatic control alone.

Guan et al. provide valuable insight into the genetic and immunological complexity of Netherton syndrome, a rare and challenging disorder caused by SPINK5 mutations. The identification of a novel compound mutation, a large SPINK5 deletion combined with the c.1258A>G polymorphism, broadens the known genetic spectrum of Netherton syndrome. Equally significant is the demonstration that intravenous immunoglobulin therapy can improve both clinical symptoms and immune dysregulation, restoring lymphocyte balance and reducing inflammatory cytokines. Although limited to a single patient, the findings highlight the promise of personalized immunotherapy and underscore the need for larger studies to validate these encouraging results. Xiao et al. marked an important step in understanding the systemic immune mechanisms underlying non-segmental vitiligo. Using single-cell RNA sequencing, the researchers revealed significant alterations in peripheral immune cell populations, including reduced KLRC2^+^ NK cells and FCGR3A^+^ cytotoxic CD8^+^ T cells, alongside increased STAM^+^ regulatory T cells. These findings challenge the traditional skin-centered view of vitiligo and highlight its broader immunological nature. By uncovering novel cellular signatures and pathways involved in disease progression, the research opens promising avenues for targeted therapies and precision medicine approaches in managing this complex autoimmune disorder.

Ohta et al. presented an interesting case report of a pustular psoriasis flare following COVID-19 infection in a 46-year-old woman with complications including scalp psoriasis, progressive generalized pustular psoriasis (GPP), and psoriatic arthritis, achieving partial remission with systemic corticosteroids, followed by etretinate, apremilast, and deucravacitinib, maintaining remission for 2 years. Systemic inflammation, neutrophilia, and skin biopsy confirmed the GPP features. This article hypothesizes that the molecular mechanisms underlying SARS-CoV-2 infection and vaccination may activate the IL-36 pathway, a central player in GPP pathogenesis, by stimulating innate immune receptors, such as Toll-like receptors (TLRs), thereby triggering cytokine cascades involving IL-6, TNF-α, and IL-36 via SARS-CoV-2 spike proteins. The activation of TLRs and IL-36 may explain disease exacerbation in predisposed individuals. The authors further concluded that SARS-CoV-2 infection can trigger GPP through IL-36-mediated inflammation, with implications for the timing and clinical features of infection- and vaccine-related flares, likely due to distinct immune kinetics. This case report can be helpful for clinical management, understanding manifestations, and evaluating strategies with corticosteroids, which are more common in infection-related cases, and the role of innate immune activation in GPP prognosis. Chen et al. reported a case study of a healthy male in his twenties with 12-year refractory psoriasis who developed multiple eruptive dermatofibromas (MEDFs) after sequential biologic therapies, including secukinumab, guselkumab, and adalimumab, as a therapeutic strategy. Physical examination revealed erythematous plaques and multiple firm, reddish-brown papules/nodules mainly on the extremities and trunk. Histological identification confirmed through fibrohistiocytic spindle cells, collagen proliferation, FXIIIa positivity, CD34 negativity, and mast cell involvement (CD117) and scattered GATA3^+^ lymphocytes, suggesting Th2 polarization. Probable drug-induced immune activation and the causality assessment (Naranjo score 7, WHO-UMC scale), highlighting induction of immune shifts, including Th2 polarization and Treg modulation in drug use. This case suggests that the cumulative effects of biologics may promote fibroblast proliferation by altering the cytokine pathway, possibly involving mast cells and Th2 cytokines. It underscores the need for vigilance and further investigation into immune-related skin reactions during biologic therapy.

## References

[1] Tembhre MK Khan WH . Vitiligo immunopathogenesis: insight of immune components and prospects of emerging immunotherapies. Cell Immun. (2025) 13:105058. 10.1016/j.cellimm.2025.105058 41435724

[2] Tembhre M Parihar A Sharma V Sharma A Chattopadhyay P Gupta S . Alteration in regulatory T cells and programmed cell death 1-expressing regulatory T cells in active generalized vitiligo and their clinical correlation. Br J Dermatol. (2015) 172(4):940-50. 10.1111/bjd.13511 25376752

[3] Ojha V Tembhre M KGupta V . Enhanced alarmin secretion exacerbates neutrophil extracellular trap (NET) formation in active psoriasis: implication of IL-33 and TSLP in driving NET formation, inflammation and oxidative stress in psoriasis. Antioxidants. (2026) 15(1):71. 41596128 10.3390/antiox15010071 PMC12837554

